# Efficacy of electroacupuncture in the treatment of peripheral neuropathy caused by Utidelone: Study protocol for a randomized controlled trial

**DOI:** 10.3389/fneur.2023.1065635

**Published:** 2023-02-09

**Authors:** Chao Lu, Guangliang Li, Dehou Deng, Rongrong Li, Xiaoyu Li, Xukang Feng, Taoping Wu, Xiying Shao, Weiji Chen

**Affiliations:** ^1^Department of Traditional Chinese Medicine, Zhejiang Cancer Hospital, Hangzhou, China; ^2^Department of Breast Medical Oncology, Zhejiang Cancer Hospital, Hangzhou, China; ^3^Department of Acupuncture and Moxibustion, The Third Affiliated Hospital of Zhejiang Chinese Medical University (Zhongshan Hospital of Zhejiang Province), Hangzhou, China; ^4^The Third Clinical Medical College, Zhejiang Chinese Medical University, Hangzhou, China

**Keywords:** breast cancer, electroacupuncture (EA), Utidelone (UTD1), chemotherapy-induced peripheral neuropathy (CIPN), peripheral neuropathy (PN), protocol

## Abstract

**Introduction:**

Utidelone (UTD1) is a new chemotherapeutic drug for recurrent or metastatic breast cancer. However, it usually leads to severe peripheral neuropathy (PN) and causes numbness of the hands and feet and significant pain in patients' life. Electroacupuncture (EA) is considered beneficial in improving PN and relieving numbness of the hands and feet. This trial aims to evaluate the therapeutic effect of EA on PN caused by UTD1 in patients with advanced breast cancer.

**Methods and analysis:**

This study is a prospective randomized controlled trial. A total of 70 patients with PN caused by UTD1 will be randomly assigned to the EA treatment group and the control group in a ratio of 1:1. The patients in the EA treatment group will receive 2 Hz EA three times a week for 4 weeks. The patients in the control group will take mecobalamin (MeCbl) tablets orally, one tablet each, three times a day for 4 weeks. The main outcome measures will be the evaluation scale of peripheral neurotoxicity of chemotherapeutic drugs according to the European Organization for Research and Treatment of Cancer Quality of Life Questionnaire-CIPN 20-item (EORTC QLQ-CIPN20) and the peripheral neurotoxicity assessment rating according to NCI CTCAE version 5.0. Secondary outcomes will be the quality of life scale according to the European Organization for Research and Treatment of Cancer Core Quality of Life Questionnaire (EORTC QLQ-C30). The results will be evaluated at baseline, post-treatment phase, and follow-up. All major analyses will be based on the intention-to-treat principle.

**Ethics and dissemination:**

This protocol was approved by the Medical Ethics Committee of Zhejiang Cancer Hospital on 26 July 2022. The license number is IRB-2022-425. This study will provide clinical efficacy data on EA in the treatment of PN caused by UTD1 and will help to prove whether EA is an effective and safe therapy. The study results will be shared with healthcare professionals through the publication of manuscripts and conference reports.

**Trial registration number:**

ChiCTR2200062741.

## Background

Recent data from the international agency for research on cancer showed that the incidence of breast cancer has exceeded that of lung cancer, with the highest incidence rate (1). The treatment of breast cancer includes surgery, chemotherapy, radiotherapy, targeted therapy, endocrine therapy, immunotherapy, and other methods (2). However, chemotherapy plays an irreplaceable role in the treatment of breast cancer because of its remarkable efficacy in systematic treatment, especially for patients with advanced breast cancer. However, a series of adverse reactions or side effects after chemotherapy should be paid greater attention. Chemotherapy-induced peripheral neuropathy (CIPN) is a common side effect of many chemotherapeutic drugs, especially in platinum compounds (e.g., cisplatin, carboplatin, and oxaliplatin), taxanes (e.g., paclitaxel, docetaxel, and albumin paclitaxel), anthracyclines (e.g., doxorubicin, epirubicin, and liposome doxorubicin), vinblastine, and other chemotherapeutic drugs (3). It is mainly manifested as limb numbness or abnormal sensation (e.g., as an ant crawling sensation and foreign body sensation), even loss of sensation or abnormal pain, and decreased tendon reflexes (4, 5). Antioxidants, neuroprotective agents or nutritive agents, and antiepileptic drugs are commonly used to treat CIPN, but they may bring other side effects or unsatisfactory efficacy (3, 6). CIPN symptoms can last for months or even years, not only affecting the progress and effect of cancer treatment but also easily causing emotional instability in patients, which seriously affects their life quality.

Utidelone (UTD1) is a new chemotherapeutic drug in recent years. It is a novel microtubule stabilizing agent and is an epothilone B analog that was produced by genetic engineering. It shows a wide range of antitumor activities in solid tumors (7, 8). At present, UTD1 is mainly used to treat recurrent or metastatic breast cancer (9), especially in patients who need high-dose anthracycline and taxus drugs or who develop resistance to them (10, 11). Some studies have shown that patients with advanced breast cancer who used UTD1 combined with capecitabine chemotherapy can prolong the 4-month survival time compared with capecitabine chemotherapy alone (10, 12). However, the main side effect of UTD1 is severe peripheral neuropathy (PN) (8). It can cause patients severe numbness of hands and feet, and some patients also feel discomfort such as sore limbs and pain. According to the literature, the incidence of PN caused by UTD1 combined with capecitabine is 85.4%, and the incidence of grade 3 CIPN is as high as 25.1%. Compared with capecitabine alone, the incidence of CIPN is 9.2%, and the incidence of grade 3 CIPN is <1% (10). Therefore, UTD1 can more easily lead to severe PN. Severe PN caused by UTD1 may reduce or withdraw from chemotherapeutic drugs and affect clinical efficacy. However, at present, there are few prevention and treatment schemes for severe PN caused by UTD1. Only one study pointed out that (11) ganglioside monosialic acid GM1 may reduce the severe PN induced by UTD1 plus capecitabine in metastatic breast cancer, but further research is still needed. Therefore, there is an urgent need for a treatment scheme that can effectively alleviate PN caused by UTD1 to improve the quality of life of patients with advanced breast cancer.

Acupuncture, as an important part of traditional Chinese medicine, has played an essential role in treating cancer-related diseases, especially in preventing and treating the side effects of chemotherapy (13, 14). Some studies have shown that acupuncture could effectively treat PN caused by various diseases, improve peripheral nerve conduction velocity (NCV), and alleviate patients' symptoms such as numbness, pain, or paresthesia of limbs (15). Acupuncture is also considered effective and safe in treating CIPN (16), with a better overall curative effect than that neurotrophic drugs (17). Electroacupuncture (EA) is a new acupuncture technology that combines traditional acupuncture technology with electrical stimulation, which has been widely used in treating nervous system diseases. Many studies showed that EA therapy could effectively treat CIPN and relieve numbness or pain in the hands and feet caused by chemotherapeutic drugs (18, 19). Animal experiments showed that low-frequency EA was more effective than high-frequency EA in alleviating CIPN symptoms (20). Stimulation with 2 Hz EA could reduce the mechanical pain and hyperalgesia of CIPN induced by paclitaxel in rats (21, 22) and also had a pronounced therapeutic effect on CIPN induced by oxaliplatin in rats (23). However, there is no study report about the efficacy of EA in the treatment of PN caused by UTD1. Whether EA can alleviate the numbness and pain in the hands and feet caused by UTD1 is unknown. Therefore, we designed a pilot RCT to evaluate the efficacy of EA on PN caused by UTD1.

The research will be a pilot clinical study. As there is no effective data on the study of EA treatment of CIPN, and considering that UTD1 is newly listed in China, only one of our cancer hospitals can use it in the whole province. We hope to collect as many cases as possible, but considering that there is a lack of large sample patients in the clinic, not all patients using UTD1 can be included in this study. We cannot set up a large sample size of controlled studies or multicenter studies. Therefore, we preliminarily plan to collect 35 cases in each group according to the minimum sample size of the clinical prospective trial.

## Methods

### Objective

The objective of this study was to evaluate whether EA will be an effective and safe therapy for PN caused by UTD1.

### Hypotheses

1. Electroacupuncture can improve PN symptoms caused by UTD1 compared with drug intervention. Subjective outcomes such as the evaluation scale of the European Organization for Research and Treatment of Cancer Quality of Life Questionnaire-CIPN 20-item (EORTC QLQ-CIPN20) can be improved.

2. EA can be considered a safe therapy in the treatment of advanced breast cancer. EA can improve a patient's life quality while treating PN caused by UTD1, as measured by the European Organization for Research and Treatment of Cancer Core Quality of Life Questionnaire (EORTC QLQ-C30).

### Study design

**Figure 1:**
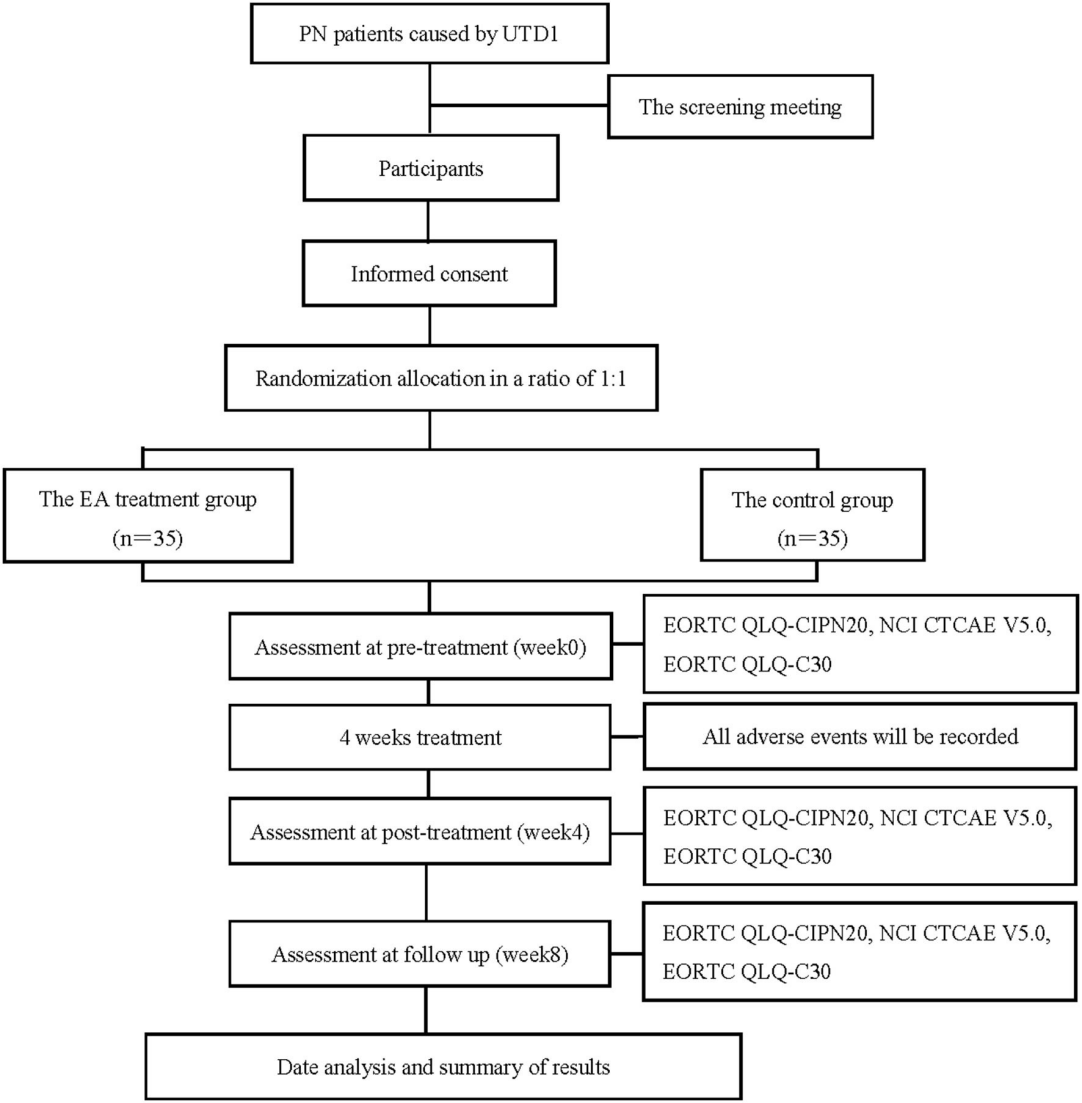
Flow chart of the study process. PN, peripheral neuropathy; UTD1, Utidelone; EA, Electroacupuncture; EORTC QLQ-CIPN20, the European Organization for Research and Treatment of Cancer, Quality of Life Questionnaire-chemotherapy-induced peripheral neuropathy 20; NCI CTCAE V5.0, National Cancer Institute Common Terminology Criteria for Adverse Events Version 5.0; EORTC QLQ-C30, the European Organization for Research and Treatment of Cancer, Quality of Life Questionnaire-Core 30.

The trial is designed as a prospective, randomized, controlled, and blind study to compare the therapeutic effects of EA therapy on PN caused by UTD1. A total of 70 participants with PN caused by UTD1 will be randomly divided into two groups in a ratio of 1:1, which are namely the EA treatment group and the control group. Figure 1 shows the flowchart, which describes the research flow in detail. In addition, the trial schedule of enrolment, treatments, and outcome assessments is shown in Table 1. The reporting of this protocol is based on the SPIRIT reporting guidelines (24).

**Table 1:** Schedule of enrolment, treatments, and assessments.

**Study period**	**Screening**	**Baseline, week 0**	**Treatment period, week 1–4**	**Post-treatment, week 4**	**Follow-up, week 8**
**Enrolment**					
Eligibility screening	•				
Informed consent	•				
Random allocation	•				
Treatment			•		
**Outcome assessment**					
(1) EORTC QLQ-CIPN20		•		•	•
(2) NCI CTCAE V5.0		•		•	•
(3) EORTC QLQ-C30		•		•	•
Safety assessment		•	•	•	•

•, Required; EORTC QLQ-CIPN20, the European Organization for Research and Treatment of Cancer, Quality of Life Questionnaire-Chemotherapy-induced peripheral neuropathy 20; NCI CTCAE V5.0, National Cancer Institute Common Terminology Criteria for Adverse Events Version 5.0; EORTC QLQ-C30, the European Organization for Research and Treatment of Cancer, Quality of Life Questionnaire-Core 30.

### Ethical standards and registration

The trial will be conducted in accordance with the principles of the Declaration of Helsinki. This protocol was approved by the Medical Ethics Committee of Zhejiang Cancer Hospital on 26 July 2022 (approval number: IRB-2022-425) and registered in the Chinese Clinical Trial Registry (chictr.org.cn) with the identification number ChiCTR2200062741.

### Informed consent

All patients with PN caused by UTD1 participating in the trial will have the right to obtain all relevant information about the trial, including any benefits or potential risks, and they can participate according to their own wishes. All participants will be allowed to drop out at any point of the trial due to their wishes. The information about participants will be kept confidential. All participants need to provide written informed consent if they are included in the trial.

### Study procedure

The duration of the trial will include three study phases, including screening in the baseline phase (week 0), 4-week treatment phase (weeks 1–4), and follow-up phase (week 8). The qualification of prospective participants will be determined by researchers who will not be involved in the assessment or treatment. At the screening meeting, patients will be given more detailed information about the study procedures. Before the first treatment (baseline, week 0), after the last treatment (post-treatment, week 4), and in the fourth week of follow-up after treatment (week 8), all participants will be required to complete several questionnaires as the outcomes of the study to evaluate the curative effect, including EORTC QLQ-CIPN20 and EORTC QLQ-C30 questionnaires. The participants cannot take drugs that may influence the outcomes of the study during the trial, including pain drugs or unspecified CIPN-related therapeutic drugs. If the patient has to take drugs that will influence the outcomes of the study due to disease progression, the patient will be considered to have dropped out. To promote enrollment, all treatment costs and outcome measurements will be free for participants.

### Participants and recruitment

Participants with PN caused by UTD1 will be recruited at Zhejiang Cancer Hospital between 1 September 2022 and 30 June 2024. Recruitment posters will be placed in the department of breast medical oncology to increase exposure.

### Inclusion criteria and exclusion criteria

All participants will be screened according to the inclusion criteria and exclusion criteria at the baseline (Table 2 for details).

**Table 2:** Inclusion criteria and exclusion criteria.

**Patients who meet the following requirements will be considered for inclusion:**	**Patients will be excluded if they have:**
(1) Diagnosed as advanced breast cancer and PN symptoms occur after using UTD1. The patient had no obvious PN symptoms before using UTD1; (2) Over 18 and under 70 years old, and the expected survival time was ≥6 months; (3) Have daily living ability and can complete all questionnaire investigation; (4) No serious heart, brain, kidney, and other diseases, KPS score >70; (5) Clear consciousness without severe mental illness or cognitive impairment; (6) Sign the written informed consent form.	(1) Diagnoses as other severe systemic diseases (e.g., cardiovascular disease, acute infectious disease, hematopathy, et al.); (2) Diagnoses as severe mental disorders, like schizophrenia; (3) PN caused by other diseases, such as diabetic peripheral neuropathy; (4) Alcohol and/or other drug abuse or dependence; (5) Pregnant and lactating women; (6) Participating in other clinical trials.

PN, peripheral neuropathy; UTD1, Utidelone; KPS, Karnofsky performance status.

### Randomization and allocation concealment

The randomization will be performed by the Department of Scientific Research, Zhejiang Cancer Hospital. A randomization method will be used to generate the random allocation sequence of two groups in a ratio of 1:1; two groups of random serial numbers will be randomly placed in opaque, sealed envelopes by other staff who will not be involved in the study to ensure the concealment of distribution. These envelopes will be printed with consecutive numbers on the outside. The envelope with the corresponding serial number will be selected according to the participant's visit order.

### Blinding

Participants will be informed that they have a 50% chance of being assigned to receive either of the two treatments: the EA treatment or drug treatment. However, as there is a complete difference between the treatment methods of the two groups, the participants cannot be blinded. At the same time, although acupuncture doctors also cannot achieve blindness because of the need for treatment, they will not be involved in the assessments or data analyses. The follow-up data managers who collect data and outcome assessors will be unaware of the group allocation of the participants.

## Intervention

### The EA treatment group

The selection of acupoints is based on the experience of acupuncture experts and referring to previous literature on acupuncture treatment of CIPN, the high-frequency application acupoints are selected. The location standard of the acupoints is based on the WHO standard for acupuncture point location (25). The location of selected acupoints is shown in Table 3. Bilateral Quchi (LI11), Waiguan (SJ5), Houxi (SI3), Hegu (LI4), Baxie (EX-UE9), Taichong (LR3), Sanyinjiao (SP6), Yinlingquan (GB34), Yanglingquan (SP9), Zusanli (ST36), and Bafeng (EX-LE10) acupoints will be taken in the limbs. Upper limb EA will connect Quchi (LI11) with Waiguan (SJ5); lower limb EA will connect Sanyinjiao (SP6) with Yanglingquan (SP9). The EA frequency will be 2 Hz, and the stimulation intensity will be based on the patient's tolerance (no discomfort). The duration of each treatment will be 30 min, three times a week for 4 weeks.

**Table 3:** Location of acupoints for treating PN caused by UTD1.

**Acupoints**	**Location**
Hegu (LI4, bilateral)	On the back of the hand, between the first and second metacarpals, at the midpoint of the radial side of the second metacarpal.
Houxi (SI3, bilateral)	Slightly clench the fist, the distal side of the ulnar side behind the palmar joint of the fifth finger, the horizontal pattern of the palm, and the red and white flesh border of the head.
Waiguan (SJ5, bilateral)	2 cun above the transverse crease of the dorsal wrist, the midpoint of the gap between ulna and radius.
Quchi (LI11, bilateral)	Bend the elbow at a right angle when the elbow bends at the end of the transverse line.
Baxie (EX-UE9, bilateral)	There are 8 points on the back of the hand, behind the edge of the web between the first and fifth fingers, between the red and white flesh, left and right.
Zusanli (ST36, bilateral)	3 cun below genicular eye of outside, one finger -breadth (middle finger) from the anterior crest of tibia.
Yanglingquan (GB34, bilateral)	On the outside of the lower leg, when the fibular head is in the depression before and below.
Yinlingquan (SP9, bilateral)	In the depression between the medial lower edge of the lower tibia and the medial edge of the tibia.
Sanyinjiao (SP6, bilateral)	3 cun above the medial malleolus, on the posterior border of the medial aspect of tibia.
Taichong (LR3, bilateral)	In the dorsum of the foot, between the first and second metatarsals, and in the depression in front of the metatarsal junction.
Bafeng (EX-LE10, bilateral)	Between the first to fifth toes, at the red and white flesh border behind the toe web edge, there are four acupoints on one side, with a total of eight acupoints on the left and right.

Equipment: the Huatuo brand disposable acupuncture needles produced by Suzhou Medical Instrument Factory will be adopted, with models of 0.25 ^*^ 40 MM. The Hans-200A model will be adopted as EA equipment.

### The control group

The participants will be treated with oral mecobalamin (MeCbl) tablets, one tablet (0.5 mg) each, three times a day for 4 weeks.

### Outcome measures

The severity of PN caused by UTD1 will be evaluated according to the evaluation scale of peripheral neurotoxicity of chemotherapeutic drugs according to EORTC QLQ-CIPN20, the peripheral neurotoxicity grades will be evaluated according to NCI CTCAE version 5.0, and the quality of life scale according to EORTC QLQ-C30 will be the outcomes used to evaluate efficacy and safety. Adverse events (AEs) will also be recorded to evaluate safety.

### Primary outcome

#### The evaluation scale of peripheral neurotoxicity of chemotherapeutic drugs according to EORTC QLQ-CIPN20

There are 20 items in the questionnaire, including 9 items in the subscale of sensory nerve symptoms (involving tingling, numbness, pain, walking or standing instability, temperature perception, and hearing), 8 items in the motor nerve symptom subscale (involving spasm writing, manipulation of small objects, and weakness), and 3 items in the autonomic nervous symptom subscale (involving vision, vertigo after changing posture, and erectile dysfunction) (26). The scoring method is the same as that of other QLQ scales. All items are scored with four levels: “no, a little, quite, and very,” respectively, assigned 1–4 points. After standardizing the scores of each dimension according to the scoring requirements, the score range of each dimension is 0–100 points. The higher the score, the more the severe CIPN symptoms. At present, the reliability and validity of the tool have been tested to a certain extent in some countries (27, 28), and it has been applied to corresponding studies (29, 30).

#### Peripheral neurotoxicity grades will be assessed according to NCI CTCAE version 5.0

This outcome will be determined by the professional evaluator according to the symptoms of PN in the patient, including checking the degree of peripheral nerve damage of the participant, such as muscle strength and muscle tension. In grade I, slight sensory numbness or tendon reflex disappeared, with no function affected. Grade II includes moderate sensory numbness or sensory loss, which affects function but does not affect daily life. Grade III includes significant sensory numbness or sensory loss, which affects daily life. Grade IV includes sensory-motor neuropathy, which severely interferes with daily life (31).

### Secondary outcome

#### The quality of life scale according to EORTC QLQ-C30

The European Organization for Research and Treatment of Cancer QLQ-C30 is a quality-of-life instrument for use in international clinical trials in oncology (32). QLQ-C30 scale includes five functional subscales, three symptom subscales, six single symptom measurement items, and one overall health subscale. The higher the functional subscale score, the better the quality of life, and the higher the symptom subscale score, the worse the quality of life.

### Safety evaluation

All AEs during the study will be recorded, including EA-related AEs (e.g., hematoma, dizziness, and nausea) and drug-related AEs (e.g., gastrointestinal discomfort and nausea). The AEs occurrence time, treatment process, result, follow-up, and whether the participant will continue to be included in the trial will be recorded. In addition, whether the patient's cancer condition develops and quality of life will also be considered safety evaluation indicators.

### Quality control

All acupuncture doctors and evaluators will be required to undergo special training before the trial to ensure consistent practices. The training contents will include the diagnosis of PN caused by UTD1, acupoints location, EA application technology, inclusion criteria, exclusion criteria, and evaluation criteria of case report forms. All the study data will be recorded in case report forms and entered into the electronic data capture system by independent researchers. Dropouts will be recorded throughout the trial.

This trial will be monitored by the Department of Scientific Research, Zhejiang Cancer Hospital. They will be responsible for monitoring the data and verifying the authenticity between the raw data and the recorded data to guarantee accuracy and quality throughout the study.

## Statistical methods

### Sample size

This is a prospective clinical study. According to the principles of the randomized controlled trial, the EA treatment group and the control group are designed, and 35 patients will be included in each group.

### Statistical analysis

The outcome data will be analyzed by the Statistical Package for the Social Sciences (SPSS) version 26.0 statistical software package. The researchers conducting data analysis will not know the distribution of participants and treatment methods. Categorical data will be displayed as counts and percentages. Numerical data of normal distribution will be expressed as mean ± standard deviations, while data of non-normal distribution will be expressed as median, minimum value, and maximum value of 95% confidence intervals. To compare the two independent samples, the *t*-test will be used to determine the numerical data of normal distribution, while the non-parametric test will be used to determine the data of non-normal distribution. The chi-square test or non-parametric tests will be used to determine categorical data. All hypothesis tests will be bilateral, with the level of significance established at 0.05.

## Discussion

Chemotherapy-induced peripheral neuropathy is a common adverse reaction related to chemotherapeutic drugs. Approximately 50–90% of patients who receive chemotherapy will have CIPN, and 30–40% of them will develop chronic adverse reactions (33–35). For example, among patients with breast cancer receiving docetaxel treatment, 42% still had CIPN symptoms for 2 years after treatment; 2 years after oxaliplatin treatment, the incidence of CIPN in patients with colorectal cancer was 84% (36, 37). CIPN is mainly manifested as symmetrical numbness, pain, and paresthesia at the ends of the extremities. In severe cases, it may involve the proximal extremities and be accompanied by the disappearance of tendon reflex or dyskinesia. The pathogenesis of CIPN is still not clear, which may be related to the damage of sensory neurons in the dorsal root ganglion (DRG), abnormal secretion of pro-inflammatory cytokines, mitochondrial dysfunction and oxidative stress, the abnormal immune system triggered by chemotherapy, microvascular destruction and axonal degeneration, imbalance of ion channels, and others factors (3, 38–40). At present, CIPN has no specific therapeutic drug, and duloxetine is the only drug recommended in the clinical guidelines (41), but it is not widely used in clinical practice because of its side effects. Due to its high prevalence and difficulty to cure, CIPN constitutes a significant clinical problem for patients with cancer as well as their healthcare providers.

Utidelone is a new chemotherapeutic drug in recent years, which is mainly used to treat recurrent or metastatic breast cancer (9), especially in patients who need high-dose anthracycline and taxus drugs or who develop resistance to them (10, 11). However, the main side effect of UTD1 can be more severe PN symptoms. It can cause patients severe numbness in their hands and feet, sore limbs and pain, and even render them unable to walk. Severe PN caused by UTD1 may lead to the reduction or withdrawal of chemotherapeutic drugs and affect clinical efficacy. However, at present, there are very seldom prevention and treatment schemes. Although the pathogenesis of PN caused by UTD1 is not clear, its symptoms are similar to those caused by other chemotherapeutic drugs. Considering that EA therapy has a good treatment effect on PN caused by these kinds of chemotherapeutic drugs (19), we speculate that EA can also improve the symptoms of PN caused by UTD1 and alleviate the numbness and pain symptoms in the patient's hands and feet. Therefore, we designed a study protocol of a randomized controlled trial by using EA technology as the main treatment method and oral drugs as the control to treat PN caused by UTD1. The EA therapy will be used to treat PN caused by UTD1 for the first time. The results of this trial will help us determine whether EA can treat PN caused by UTD1 and improve the quality of life of patients with advanced breast cancer.

At present, there are no definite tools for evaluating the PN caused by UTD1. A numeric rating scale (NRS) for cancer pain has been applied to the clinical evaluation of various pain degrees since 1950 (42). The number between 0 and 10 is used to represent the pain intensity, which is applicable to the elderly and those with low educational levels person (43). However, due to its low sensitivity, it is rarely used to evaluate the degree of CIPN. The evaluation scale of EORTC QLQ-CIPN20 is a patient-self-reported questionnaire (26). As doctors and patients have different understandings of the disease, different evaluation results of CIPN may result. Especially when it comes to sensory disorders, there are significant differences between the two groups. Therefore, a reliable assessment of the severity of CIPN-related sensory disorders should combine clinical symptoms with a professional assessment. Peripheral NCV is commonly used to evaluate the degree of peripheral nerve damage. However, as our study subjects are patients with advanced breast cancer, NCV tests may cause discomfort to patients or increase their psychological burden. Therefore, we may recommend this test to patients who are willing, but it will not be used as the outcome of our study. In this study, the severity of PN caused by UTD1 will be evaluated using the evaluation scale of peripheral neurotoxicity of chemotherapeutic drugs according to EORTC QLQ-CIPN20 as the main outcome. To evaluate the accuracy, the professional evaluator will also comprehensively evaluate the peripheral neurotoxicity severity of the patient according to the patient's clinical symptoms and physical examination of the peripheral nervous system, and the severity grade will be determined according to NCCN5.0. In addition, the patient's quality of life will be evaluated according to EORTC QLQ-C30, and AEs during the study will be recorded, which will make the study results more convincing.

Although duloxetine is the only drug recommended by the guidelines to treat CIPN, it is rarely used in clinical practice due to the acceptance of antidepressants, adverse reactions of the central nervous system and digestive system, and its limited efficacy (duloxetine 58% vs. placebo 38%) (44). Neurotrophic drugs are widely used in the clinical treatment of peripheral nerve diseases and MeCbl is the most commonly used. Some studies showed that MeCbl can improve the numbness of hands and feet caused by all kinds of PN (45, 46) and is considered a safe, effective, and generally well-tolerated drug for treating PN (47). In clinical treatment, MeCbl is most widely used in the treatment of CIPN symptoms, which is also based on our clinical data. When reviewing the previous treatment data, it was found that seldom few patients were willing to receive drug treatment such as duloxetine, and pregabalin was used more for patients with neuralgia. Therefore, we chose MeCbl as the control drug to treat PN caused by UTD1 according to previous clinical experience. From another perspective, this study will also clarify the effectiveness and safety of MeCbl in the treatment of PN caused by UTD1.

Utidelone is mainly used for patients with recurrent or metastatic advanced breast cancer. These patients mainly have accumulated neurotoxicity of chemotherapeutic drugs in their bodies because they previously used large doses of anthracycline and taxus drugs. UTD1 may induce neurotoxicity accumulated by other chemotherapeutic drugs, or it may itself be a pathogenic drug that causes severe PN symptoms, but in either case, the patients who developed PN symptoms are related to UTD1, thus our research theme is consistent. The severity of PN symptoms caused by UTD1 is closely related to drug dose accumulation. The PN symptoms of patients who used UTD1 in the first few times are usually not serious, and these patients' symptoms can be relieved during the interval of chemotherapy, so they usually do not want other interventions. However, with the increase in chemotherapy times, the PN symptoms of patients become more serious. Their symptoms cannot be relieved during the chemotherapy interval, and they feel numbness and discomfort at the end of the limbs all the time. PN caused by UTD1 is mainly characterized by numbness, tingling, or other abnormal feelings in the hands and feet. Generally, the first symptom that patients experience is sensory nerve damage. However, patients who have used chemotherapy for UTD1 often develop motor nerve function damage. In particular, patients have weakness in both lower limbs and difficulty walking, some patients may also have muscle atrophy, but autonomic nerve function is rarely affected. It is highlighted here that sensory nerve symptoms are the most common in patients. Therefore, patients with only sensory nerve symptoms, such as patients with a certain degree of numbness, will be included in this study. During the recruitment, the doctors will evaluate the symptoms of PN according to the patient's condition. For the patient's simple numbness of individual fingers, perhaps the symptom of nerve compression, we will identify it clinically.

This study will clarify the efficacy of EA in the treatment of PN caused by UTD1 and provide technical guidance for clinical treatment. The results will further provide new clues, new ideas, and new methods for acupuncture in the treatment of nervous system diseases and cancer-related diseases. Finally, we hope the results of this study will effectively improve the PN symptoms caused by UTD1 in patients with advanced breast cancer, reduce their pain, and improve their quality of life.

## Ethics statement

Ethical approval was granted on 26 July 2022 by the Medical Ethical Committee of Cancer Hospital of the University of Chinese Academy of Sciences (Zhejiang Cancer Hospital). The license number is IRB-2022-425. The patients/participants provided their written informed consent to participate in this study.

## Author contributions

CL, WC, and GL contributed to the study's design, drafting, and editing of the manuscript. CL wrote the first manuscript for the trial and edited the final manuscript. GL and XS revised the manuscript. XF and TW evaluated participant information and collected CRF data. DD conducted the EA treatment. RL registered the study result data. XL analyzed the data. All authors read and approved the final manuscript.
